# Supplementary material for: Stomatin encapsulates aquaporin-1 and urea transporter-B in the erythrocyte membrane
Source: bioRxiv. 2025 Aug 29:2025.08.29.673128. Preprint. [Version 1] doi: 10.1101/2025.08.29.673128 (PMC12407830; doi:10.1101/2025.08.29.673128)
Supplement: Supplement 1 [file NIHPP2025.08.29.673128v1-supplement-1.pdf]

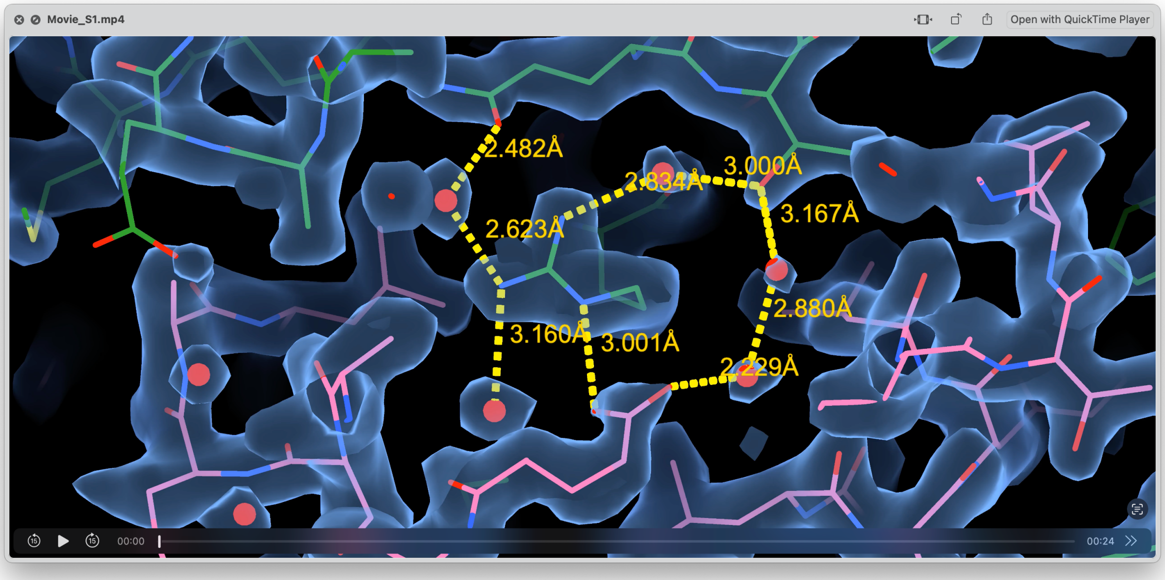

Movie S1: Hydrogen-bonded network of waters between the CC2 domain and the C-terminal beta barrel

Link

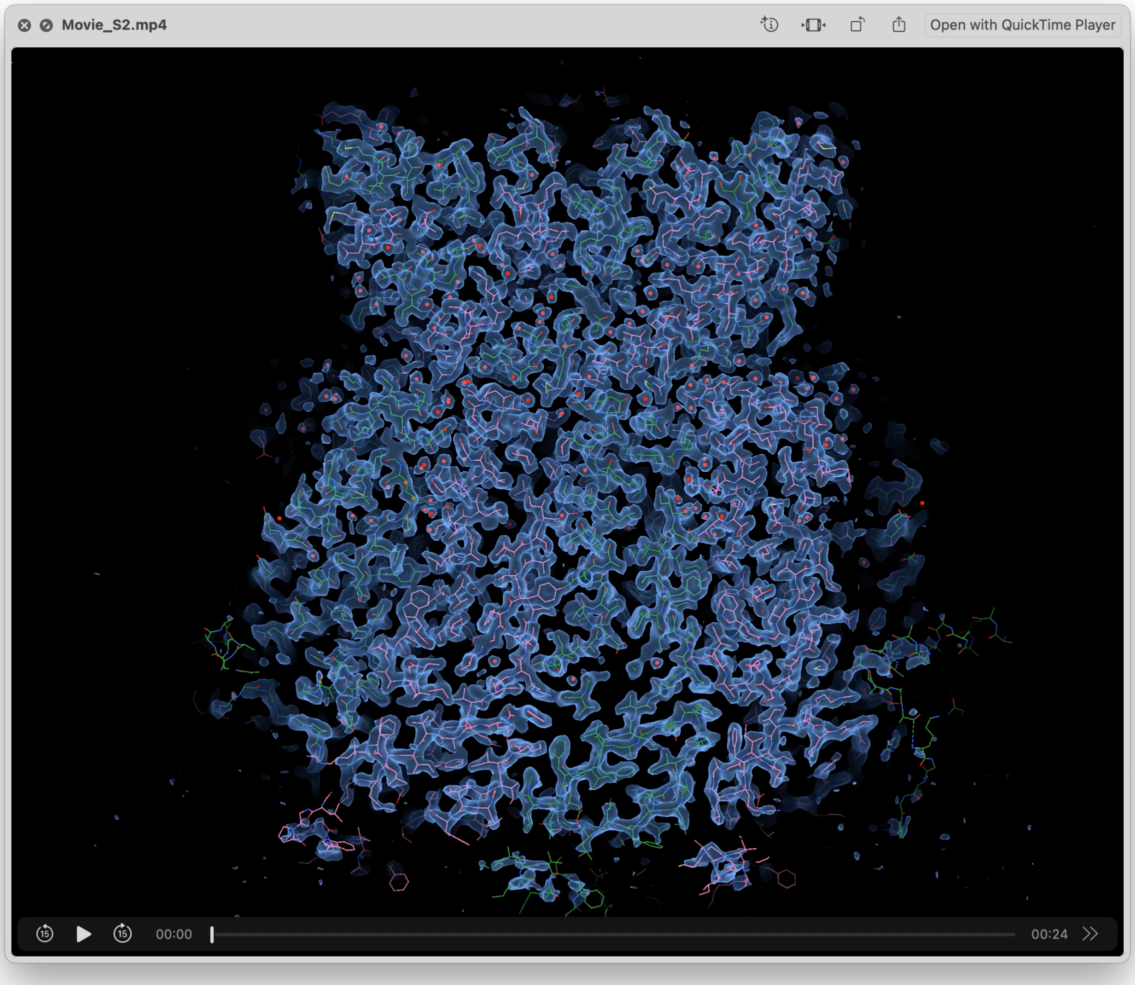

Movie S2: Overall map/model fit (C8 sharpened map)

Link
